# Distinct factors drive the progression of tau pathology in Alzheimer’s disease

**DOI:** 10.1016/j.fmre.2025.04.001

**Published:** 2025-04-09

**Authors:** Yifan Luo, Honglu Yu, Keqiang Ye

**Affiliations:** a Faculty of Life and Health Sciences, Brain Cognition and Brain Disease Institute, Shenzhen Institutes of Advanced Technology, Chinese Academy of Sciences, Shenzhen 518055, China; b Faculty of Life and Health Sciences, Shenzhen University of Advanced Technology, Shenzhen 518055, China

**Keywords:** Alzheime’s disease, Tau pathology, Neurofibrillary tangles, Neurodegenerative diseases, Cognitive impairment

## Abstract

Alzheimer’s disease (AD) is the most common cause of dementia worldwide. The primary histopathological markers for AD diagnosis are extracellular amyloid plaques and intracellular neurofibrillary tangles (NFTs), featured by aggregation of hyperphosphorylated and truncated tau proteins. Emerging evidence shows that tau pathology, rather than amyloid-β deposition, exhibits a stronger correlation with brain atrophy and cognitive decline in AD, emphasizing its pivotal role in disease progression. However, the molecular mechanisms of tau propagation in the brain are incompletely understood, and there is no effective therapy to halt tau pathology propagation in AD. In this review, we summarize current knowledge on the multifactorial triggers of tau pathology in AD in the aspects of (1) physiological or pathological driving factors, (2) different types of brain cells and (3) key regulatory proteins that steer tau aggregation and spread. Based on these findings, we also critically evaluate the current and potential therapeutic strategies against tau pathology in AD. Together, this review provides a comprehensive understanding of tau pathology regulation and highlights promising strategies for therapeutic intervention.

## 1 Introduction

In 2023, over 55 million people worldwide are suffering from Alzheimer’s disease (AD) and other forms of dementia, with the number projected to double every 20 years. Neurofibrillary tangles (NFTs) composed of hyperphosphorylated and cleaved tau proteins and amyloid plaques of the amyloid-β (Aβ) are two major pathologies in AD. Tau (gene name: *MAPT*, microtubule-associated protein tau) is predominantly expressed in neurons as part of the microtubule network. Although encoded by a single gene *MAPT*, tau has six different isoforms by alternative splicing [1]. The major difference among these isoforms in tau pathology is the number of microtubule-binding repeat domains (3R/4R). The ratio of 4R/3R tau alters the properties of tau aggregation and pathologies. 4R tau, containing one extra repeat domain, generally exhibits a higher propensity for aggregation compared to 3R tau. In a healthy human brain, 4R tau and 3R tau are expressed at almost a 1:1 ratio. However, the imbalanced ratios are found in different tauopathies, including AD. Both primary 3R NFTs and primary 4R NFTs exist in different tauopathies. 4R tau is enriched in PSP (Progressive supranuclear palsy) and CBD (corticobasal degeneration), whereas 3R tau is enriched in Pick disease. AD, chronic traumatic encephalopathy (CTE), and primary age-related tauopathy (PART) exhibit mixed 3R/4R pathology. With over 80 phosphorylation sites identified, phosphorylation is the best-studied post-translational modification of tau. The hyperphosphorylation of tau could decrease its association with microtubules and increase its self-aggregation [1]. In AD, the hyperphosphorylated tau aggregates act as “seeds” to induce endogenous tau misfolding in neurons in a “prion-like” manner, leading to neuronal dysfunction and neurodegeneration.

Importantly, mounting clinical evidence also indicates that tau accumulation, rather than amyloid deposition, correlates with the progression of brain atrophy and cognitive dysfunction in AD. Other tauopathies (e.g., primary age-related tauopathy (PART)) do not accumulate amyloid plaque but lead to neurodegeneration, indicating that tau pathology alone is sufficient to induce neurodegeneration. Strikingly, recent reports describe PSEN1 mutation carriers harboring APOE3 R136S or RELN H3447R variants who exhibit elevated Aβ levels but markedly attenuated tau accumulation and limited neurodegeneration, challenging the amyloid cascade hypothesis and reinforcing tau’s central role in AD pathogenesis [2]. However, the molecular mechanisms underlying the progression of tau propagation across the human brain remain unclear. There is no drug available to effectively inhibit or clear tau accumulation. This review systematically examines multidimensional drivers of tau pathology in AD. These factors include: (1) physiological or pathological driving factors (aging, Aβ, diabetes mellitus, gender); (2) cell-type-specific contributions (neurons, microglia, astrocytes, non-resident immune cells); (3) key steering proteins (APOE, TREM2, Tau kinases, Tau receptors, AEP, C/EBPβ). Finally, we also emphasize the current and potential therapies to treat tau pathology in AD.

## 2 Physiological or pathological driving factors for tau pathology in AD

### 2.1 Aging

Age is the predominant risk factor for AD as well as other tauopathies. > 90% of AD patients are diagnosed after the age of 65. Beyond age 65, the risk of AD doubles every five years and rises to about 30% by the age of 85. However, aging is a complex process, and how age drives the progression of tau pathology is not well understood. Aging is characterized by several hallmarks, including genomic instability, chronic inflammation, reactive oxygen species accumulation, telomere attrition, epigenetic alterations, loss of proteostasis, impaired macroautophagy, deregulated nutrient sensing, mitochondrial dysfunction, cellular senescence, stem cell exhaustion, altered intercellular communication, and dysbiosis. These factors collectively contribute to the onset of AD pathologies through diverse mechanisms. Key mechanistic link between aging and tau pathology in AD is chronic systemic inflammation, which is a central feature of aging. Although acute inflammation may have neuroprotective functions, chronic inflammation strongly promotes the development of AD. Neuroinflammation appears to act as a leading cause for tau phosphorylation and amyloid plaque formation in AD, highlighting the important role of microglia in tau pathology, which is the major resident macrophage in the brain. The senescent glial cells also promote the hyperphosphorylation of tau proteins, indicating that cellular senescence may be an important candidate mechanism for the transition of normal aging into AD. Eliminating senescent astrocytes and microglia (identified as cells positive for p16INK4a) in Tau P301S mice leads to a decrease in NFT formation and inflammation in the brain, ultimately improving cognitive functions [3]. Apart from the chronic inflammation, DNA methylation, a well-known epigenetic modification in AD that adds methyl groups to the cytosine of DNA, has also recently become a hallmark of aging. Several DNA methylation regions (ANK1, BIN1, RHBDF2, etc.) are associated with the onset of AD [4]. Aging promotes tau pathology in AD by DNA methylation. DNA methyltransferase DNMT3A2 expression in the hippocampus decreases with age, and overexpression of DNMT3A2 restores cognitive functions in aged mice [5]. Additionally, some important proteins, which exhibit key functions on tau spreading, also increase with aging. For example, C/EBPβ/AEP signaling (discussed below) drives AD progression by different mechanisms [6]. Undoubtedly, more effort is required to delineate the mechanism of how aging drives tau pathology. In addition, Primary age-related tauopathy (PART) is a neurodegenerative disease with cognitive impairment caused by NFTs independently of amyloid plaques. The development of PART is also found to be closely associated with age. Although the disease was only recently classified in 2014 and the data remain limited, most of the reported patients are over 60 years old, further indicating that tau accumulation could be strongly driven by aging.

### 2.2 Amyloid-β (Aβ)

Aβ pathology potently accelerates the initiation and propagation of tau pathology. Strong clinical evidence emerges from studies of familial AD patients harboring pathogenic mutations in APP (amyloid precursor protein), PSEN1, or PSEN2 (presenilin 1/2), which elevate Aβ production [7]. Virtually all carriers develop tau pathology and cognitive impairment at middle age. Regardless of familial or sporadic AD, tau tangles typically emerge decades after Aβ dysregulation during the preclinical phase, based on clinical positron emission tomography (PET) results [2]. NFTs first appear in the transentorhinal cortex and then spread to the hippocampus, other cortical regions and ultimately almost all regions of the brain, following the spatiotemporal tau spreading pattern in AD. Contrastingly, in primary age-related tauopathy (PART)—which lacks Aβ plaques—tau propagation progresses more slowly and remains confined to limbic regions, whereas Aβ-rich neocortical regions in AD exhibit accelerated neocortical tau dissemination. This indicates the complexity of tau pathology is the final consequence of multiple different driving factors, varying from different brain regions [7]. Although not fully understood, Aβ plaques are reported to promote tau pathology via distinct pathways. Aβ aggregation elevates the expression of several key tau kinases (CDK-5, GSK-3β, etc.), thereby facilitating tau hyperphosphorylation [8]. Aβ promotes tau aggregation by activating AEP, caspase-3, or calpain-1 to produce more aggregable tau-truncated fragments, and also triggers tau hyperphosphorylation and aggregation by stimulating neuroinflammatory responses [6,8]. In addition, Aβ plaques further disrupt mitochondrial functions and impair axonal transport to facilitate tau aggregation and neurodegeneration. Similarly, other protein aggregations are also reported to facilitate tau spreading. These misfolded proteins include α-Synuclein and TDP-43, which are the major pathological proteins in Parkinson’s disease (PD) and Amyotrophic lateral sclerosis (ALS), respectively.

### 2.3 Diabetes mellitus

Type 2 diabetes mellitus (T2DM) is a major risk factor for AD, with an odds ratio of 1.3–1.9. T2DM, characterized by hyperglycemia and insulin resistance, is a major global public health challenge. Based on the close relationship between AD and T2DM, AD is also named type 3 diabetes mellitus (T3DM). Despite accounting for only 2% of body weight, the brain consumes > 25% of the body’s total glucose. Insulin signaling is essential for neuronal development and memory formation in the brain, however, hippocampal insulin signaling and glucose metabolism start to decrease during the preclinical period of AD. In addition, 80% of AD patients develop insulin resistance or T2DM. T2DM also shares some AD-like pathological features. Similar to the AD brain, tau is presented in human pancreatic β cells, with significantly higher levels in T2DM patients. Several studies have reported direct regulation of tau phosphorylation through insulin signaling. In the brain, insulin activates phosphatidylinositol 3-kinase (PI3K) via insulin receptor substrates (IRS-1). PI3K then phosphorylates AKT that inactivates a key tau kinase—GSK-3β (glycogen synthase kinase 3β) via phosphorylation [9]. These findings suggest that tau hyperphosphorylation is promoted when insulin pathway is impaired in diabetic patients. Similarly, insulin signaling is required to suppress numerous tau kinases, including pJNK (phospho-c-Jun N-terminal kinase), pMAPK (phospho-mitogen-activated protein kinase) and GSK-3β [10]. On the other hand, tau phosphatases such as PP2A (protein phosphatase 2A) are inhibited in the in vivo diabetic models. GLP-1R agonists, which are FDA-approved drugs to treat T2DM and obesity, also show potential therapeutic effects on AD. Several animal studies demonstrated that GLP-1R agonists decrease Aβ/tau pathology, inflammation, synaptic plasticity deficits (e.g., LTP impairment). For example, Cognitive decline in patients receiving dulaglutide slows by 14% [11]. Thus, GLP1-R agonists could be further modified to potentially treat AD.

### 2.4 Gender (Woman)

Approximately 70% of AD patients are female, and they face a twofold higher lifetime risk (1 in 5) compared to men (1 in 10). Females exhibit a fourfold higher incidence of AD, with earlier onset (ages 65–75). Moreover, they tend to exhibit more severe cognitive symptoms, including a higher density of neurofibrillary tangles (NFTs) and more pronounced cognitive impairment. Women have higher burdens of tau in the brain than the men, and transcriptome-wide interaction analyses suggest sex-modulated tau phosphorylation could explain the increased risk of women to AD. Nevertheless, the mechanism of this gender disparity is not fully discovered. One important explanation involves gonadal hormones, particularly follicle-stimulating hormone (FSH) and luteinizing hormone (LH), which accumulate dramatically in postmenopausal women. For example, FSH is reported to promote neuronal tau accumulation and amyloid-β plaque formation in postmenopausal women by activating C/EBPβ/AEP pathway, which could be blocked by an FSH inhibitory antibody [12,13]. Estrogen deficiency after menopause also increases the incidences of AD in women by different mechanisms, such as dysregulation of intracellular calcium signaling and disinhition of Daxx (death-domain-associated protein) and glutamate excitotoxicity [14]. Moreover, sex chromosomes are involved in tau pathology progression. Chromosome X-linked gene encoding ubiquitin specific peptidase 11 (USP11) augments pathological tau aggregation via tau deubiquitylation initiated at lysine-281. Genetic elimination of USP11 in a tauopathy mouse model preferentially protects females from acetylated tau accumulation and cognitive impairment. Interestingly, USP11 levels also strongly associate positively with tau pathology in females but not males. Hence, X-linked USP11 increases tauopathy vulnerability in women [15]. Additionally, Sex-dependent mitochondrial dysfunction and metabolic remodeling to glycolysis in the brain are found in female AD mice, but not male counterparts, to drive AD progression [16]. Additionally, epigenetics and educational disparities may also contribute to the sexual dimorphism of AD, although the mechanisms still require further exploration.

In summary, aging, Aβ, diabetes, and sex differences are the major physiological or pathological driving factors for tau pathology in AD (Table 1). Chronic inflammation is the major hallmark of aging, strongly promoting neuroinflammation and tau phosphorylation. Aβ triggers tau hyperphosphorylation and truncation, thus promoting tau aggregation. Insulin signaling is essential for inhibition of tau phosphorylation, however, insulin signaling is impaired for diabetes patients. In addition, these factors interplay with each other and drive tau pathology as a whole network. For example, aging could also increase Aβ production and risk of diabetes, thereby promoting tau pathology.

**Table 1:** **Overview of different driving factors for tau pathology in AD**.

Driving factor types	Factor name(s)	Effect on tau pathology	Key mechanism(s)	References
Physiological or pathological driving factors	Aging	Promote	Chronic inflammation; DNA methylation; etc	[4]
	Aβ	Promote	Increase tau phosphorylation and truncation	[7,8]
	Diabetes mellitus	Promote	Disinactivate tau kinases	[9]
	Woman	Promote	C/EBPβ/AEP pathway activation	[12,13]
Different brain cell types	Neuron	Promote	Produce and spread pathological tau	[77]
	Microglia	Promote	Tau spreading and inflammation	[17,78]
	Astrocyte	Both inhibit and promote	Elusive	[23]
	Non-resident immune cells	(Cytotoxic T cell) promote; others unknown	Neuroinflammation	[20,25]
Key proteins	APOE	Promote, especially APOE4	Tau phosphorylation in neuron and microglia disorders	[28]
	TREM2	Promote	Microglia overactivaton	[45,46,47]
	Tau kinases	Promote	Promote tau phosphorylation	[49]
	Tau receptors	Promote	Promote tau spreading	[51]
	E3 ubiquitin ligases	Inhibit	Promote tau degradation	[53,54]
	AEP	Promote	Promote tau truncation and aggregation	[59,60]
	C/EBPβ	Promote	Upregulate AEP and inflammation	[6]

## 3 Functions of different brain cell types in tau pathology in AD

### 3.1 Neuron

Intracellular tau aggregation in neurons is central pathological driver for neurodegeneration in AD patients. Under pathological conditions, hyperphosphorylated tau protein disassociates from microtubules, promoting tau self-aggregation into insoluble forms. Tau aggregation inside neurons exerts neurotoxic effects by both loss of function of supporting microtubules and gain of neurotoxic functions. The presence and the number of hyperphosphorylated tau are correlated with memory deficits. Surprisingly, neurons with NFTs may live for decades without significant structural and functional changes. Conversely, tau oligomers—not NFTs—directly mediate synaptic dysfunction and memory loss in mouse models. Moreover, tau oligomers but not PFFs (pre-formed fibrils) exert neurotoxicity in primary cultured neurons, suggesting tau oligomer is the major tau toxic form. However, the precise mechanisms of how intracellular tau leads to neuronal dysfunction are not fully understood.

**Fig. 1:**
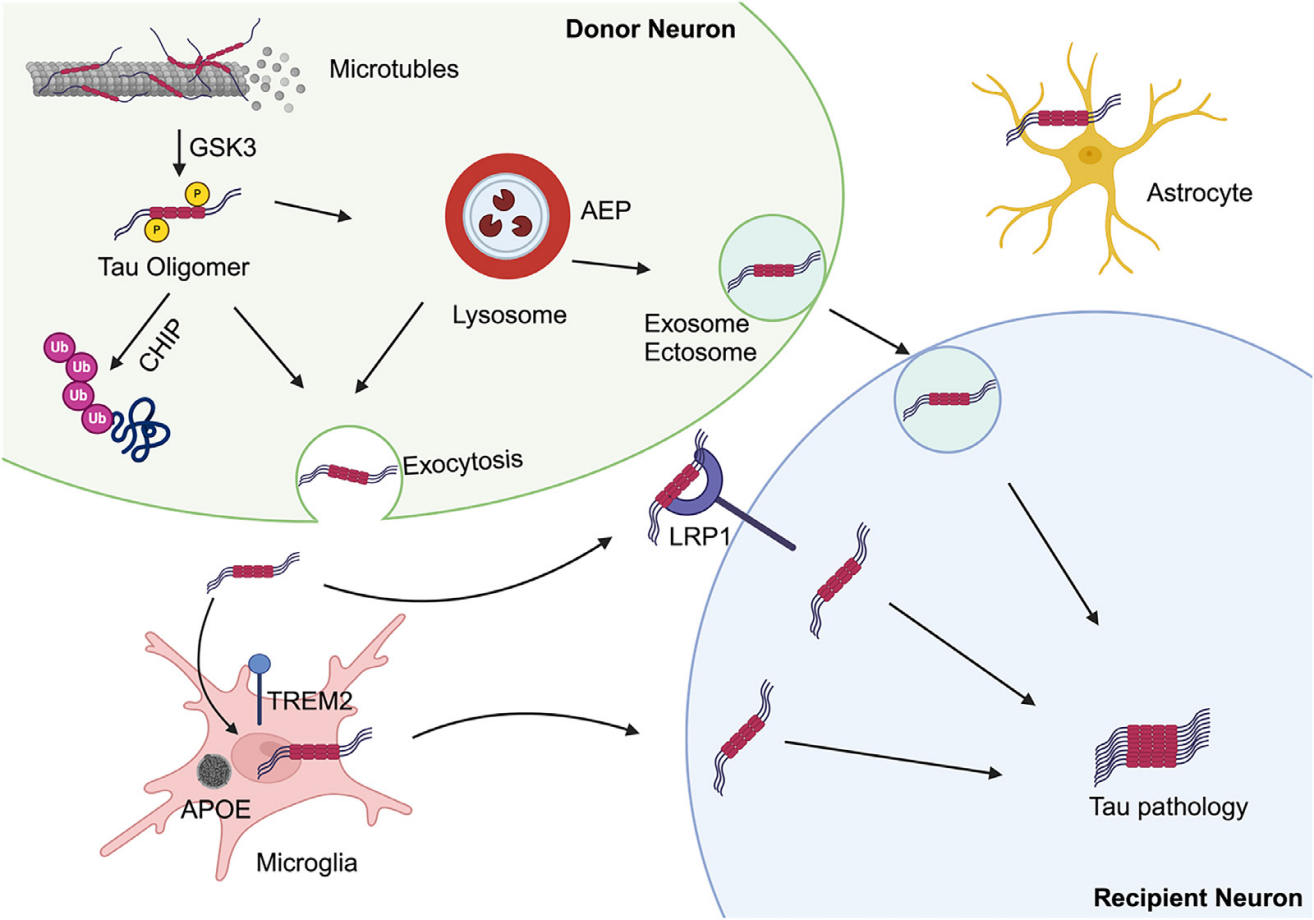
**Tau pathological spreading process in AD.** Pathological tau seeds could be generated via active AEP truncation and subsequent phosphorylation by tau kinases such as GSK3. If phosphorylated tau seeds escape from ubiquitylation by E3 ligases like CHIP, they could spread to other neurons by direct neuron communications. Moreover, pathological tau seeds could also release and then uptake via tau receptors in the recipient neurons. These conventional and unconventional protein secretion pathways include lysosomal exocytosis, synaptic vesicle release, ectosomes, exosomes, direct translocation, etc. Microglia are also activated in the AD brain through TREM2 and APOE to accelerate tau spreading and neurodegeneration. However, the functions of astrocytes in tau pathology still require further studies. This schematic was created using Biorender (http://biorender.com).

Pathological tau propagates trans-synaptically through neuron-to-neuron transmission or via extracellular release and uptake. Aggregated before or after secretion, it could then be endocytosed into the recipient neurons thereby inducing endogenous tau aggregation and toxicity there [1]. Intracellular pathogenic tau can propagate by direct neuron-to-neuron communications or release and uptake by recipient neurons. Tau spreads within connected anatomical neuronal networks and is enhanced by neuronal activities. Oligomeric tau could spread between synaptic neuronal connections at synapses. Additionally, direct attachment by tunneling nanotubes could be a potential way for direct neuron-to-neuron tau spreading. On the other hand, pathological tau seeds could also be secreted from phosphorylated tau-contained neurons. Apart from dying neurons, tau, without any signal peptides, is reported to be released from donor cells by conventional and unconventional protein secretion pathways, which includes lysosomal exocytosis, synaptic vesicle release, ectosomes, exosomes or direct translocation **(**Fig. 1**).** Furthermore, neuron engulfment by reactive microglia may release pathological tau [17]. Extracellular tau itself also shows toxicity to neurons by additional mechanisms. Diaz-Hernandez et al. reported that stimulating SH-SY5Y cells with extracellular tau triggers intracellular calcium flux by activating muscarinic receptors, resulting in cell death [18]. Notably, Tau oligomers also inhibit long-term potentiation (LTP) in the hippocampal slices. To conclude, pathological tau aggregation damages the function of neurons, and also possibly spreads to other cells in different ways. Other cell types in the brain are also importantly involved in the process and are discussed in the following parts.

### 3.2 Microglia

Microglia are the major resident macrophages in the central nervous system (CNS), accounting for 5%–15% of total cells in the human brain. The major functions of microglia in the healthy brain include synapse pruning, injury repair, homeostasis maintenance, phagocytosis, supporting and communicating with other cells. In AD and other neurodegenerative diseases, microglia transition into activated states, marked by proliferation, morphological changes, and altered gene expression. Among these active forms of microglia, disease-associated microglia (DAM) draws increasing attention. They are highly correlated to Aβ/Tau pathology and enriched in different AD/Tau mouse models and the AD patient brains [19,20]. Moreover, most of the common genetic risk factors for AD are either preferably or highly expressed in microglia, indicating the essential role of microglia in AD progression. Microglia also uptake tau via receptors (e.g., LRP1, HSPGs, LDLR) or chemokine signaling (CX3CR1, P2RY12). Before the establishment of methods enabling microglial depletion in the brain (e.g. PLX3397), it was previously believed that microglia could possibly inhibit tau spreading by phagocytosing and degrading soluble and insoluble tau. However, a landmark study by Asai et al. [21] surprisingly shows that depletion of microglia halts tau spreading in different mouse models. They also reported that microglia could facilitate tau propagation by releasing tau-containing exosomes. Recently, more and more studies have confirmed that microglia accelerate tau spreading by different mechanisms **(**Fig. 1**)** [17]. Brelstaff et al. [17] reported that microglia could phagocytose the AT8 positive neurons and turn to hypophagocytic phenotype to release tau as well as other matrix remodeling enzymes. Chronically activated microglia also drive neuroinflammation by releasing proinflammatory cytokines (TNF-α, IL-1β, IL-6 and NF-κB) and reactive oxygen species. Inhibition of either of them could alleviate neuroinflammation and neurodegeneration. Microglia can also be activated by dysbiosis (gut microbial imbalance). Dysbiosis exacerbates the progression of tau pathology in AD. The mechanism could be explained by the released proinflammatory cytokines (e.g., TNF-α, IL-6), which could cross the blood-brain barrier (BBB) to activate microglia. Furthermore, microglia orchestrate multicellular toxicity by activating astrocytes and recruiting cytotoxic T cells, amplifying tau-mediated neurodegeneration [20].

### 3.3 Astrocyte

Glial cells exhibit overlapping, distinct, and interconnected roles in tau pathology. Astrocytes, the most abundant glial cells in the brain, provide structural/metabolic support for neurons, regulate synapses, and maintain the blood-brain barrier [1]. Like microglia, astrocytes are also strongly activated in various neurodegenerative diseases, including AD and PART. Activated astrocytes accelerate the progression of different neurodegenerative diseases by secreting pro-inflammatory cytokines, complement components, and reactive oxygen species. These reactive astrocytes are called “A1” neurotoxic astrocytes, while the neuroprotective astrocytes are referred to as “A2”, with A1 predominance in AD driving neuronal loss [22]. Due to the lack of adequate methods and models for specifically studying astrocyte functions in vivo, only a limited number of studies have investigated their role in tau pathology in AD. Both alleviating and exacerbating functions of astrocytes in tau pathology were reported. Martini-Stoica et al. [23] demonstrated that astrocyte-specific TFEB overexpression enhances tau phagocytosis and reduces spreading in Tau P301S mice. Importantly, reactive astrocytes are also found in age-related tau astrogliopathy (ARTAG), which is characterized by clusters of thorn-shaped and granular or fuzzy phosphorylated tau-containing astrocytes in the gray/white matter. ARTAG is not always linked to memory loss, suggesting astrocytic tau accumulation may represent a compensatory clearance mechanism in early disease. Moreover, a recent multi-cohort biomarker study (*n* = 1016) revealed that Aβ correlates with plasma phosphorylated tau levels exclusively in individuals with astrocyte reactivity, positioning reactive astrocytes as critical mediators linking Aβ to incipient tau pathology [24]. This finding suggests that astrocyte reactivity is an important upstream event linking Aβ with initial tau pathology. Activated microglia could also induce reactive astrocytes to develop into the A1 neurotoxic phenotype by secreting IL-1α, TNF-α, and C1q, thereby facilitating tau pathology and neuronal loss. These conflicting findings underscore the dualistic—and likely stage-dependent—role of astrocytes in tau pathology, necessitating the development of stage-specific astrocyte-targeted therapies to disentangle their complex contributions.

### 3.4 Non-resident immune cells

In addition to resident immune cells like microglia and astrocytes, recent findings have revealed that non-resident immune cells are also involved in tau pathology in AD. Given that little evidence was found to show their ability to penetrate BBB, Non-resident immune cells were barely studied previously. However, in AD patients, BBB integrity is disrupted by chronic neuroinflammation and proteinopathies. When the BBB is disrupted, proteins and immune cells from the blood can translocate into brain parenchyma, triggering neuroinflammation and further neuronal damage. Until recently, many adaptive immune cells, including T cells, B cells and neutrophils have been detected in the human AD brain parenchyma. Therefore, several studies focus on the functions of these non-resident immune cells [20,25]. The infiltration of T cells dramatically increases in AD post-mortem brains compared with that in normal brains. In mouse models, interestingly, it is found that tau P301S mice exhibit greater cerebral T cell infiltration than that in amyloid-β-depositing mice [20,25]. Specifically, cytotoxic CD8+ *T* cells accumulateis increased in the tau mouse model to promote tau accumulation and neurodegeneration via interferon-γ and PDCD1 signaling [20]. Additionally, cognitively impaired female APOE ε4 carriers show increased infiltration of IL-17 and IL-1 positive neutrophils. Deletion of APOE4 in neutrophils inhibits the phenotype and reduces amyloid plaques in APP/PS1 mice [26]. However, how non-resident immune cells contribute to tau pathology progression still needs to be fully characterized.

To conclude, various types of brain cells contribute to tau spreading in AD. Pathological tau seeds initiate from neurons. They show toxic effects on neurons and could be released via different mechanisms. Microglia promote tau pathology not only by uptaking and spreading toxic tau seeds but also by elevating inflammation. Moreover, activated microglia also activate astrocytes and recruit T cells to accelerate tau pathology. Both ameliorating and exacerbating functions of astrocytes in tau pathology are reported. However, the conclusive function of astrocytes can only be determined when tools for astrocyte-specific regulation are introduced.

## 4 Key proteins involving in progression of tau pathology in AD

### 4.1 Apolipoprotein E (APOE)

Apolipoprotein E (APOE) is the most significant genetic risk factor and the most extensively studied protein in AD pathogenesis. There are three predominant alleles for human APOE gene: ε2 (*APOE2*), ε3 (*APOE3*), and ε4 (*APOE4*). The *APOE4* allele strongly increases AD risk to 3–5-fold as heterozygotes and 15-fold as homozygotes. Generally, about one-fourth of the population are *APOE4* allele carriers, and 2% to 3% of people are *APOE4* homozygotes. Conversely, *APOE2* allele is protective in AD, compared with the most common *APOE3* allele. The difference between the three APOE isoforms is the cysteine or arginine amino acid at the position of 112 and 158 (APOE2: Cys112/Cys158; APOE3: Cys112/Arg158; APOE4: Arg112/Arg158). The mature form of APOE protein is a 299-aa glycoprotein after cleavage of an 18-aa signal peptide at N terminus. Astrocytes account for ∼70% of CNS APOE production, most of which is secreted. Other brain cells including microglia, neurons, vascular mural cells and choroid plexus cells are also reported to express APOE. The major function of APOE is to act as a lipid transporter of cholesterol and phospholipids by binding to its receptors at cell surfaces. These identified APOE receptors include low-density lipoprotein receptor (LDLR), LDLR-related protein 1 (LRP1), heparan sulfate proteoglycans (HSPG) and TREM2. Based on the crystal structures, functional analysis and experimental validation, APOE variants show different binding preferences towards various lipoproteins or their receptors. APOE4 exhibits preferential binding to VLDLs, whereas APOE3 binds preferentially to high-density lipoproteins (HDLs), resulting in different plasma cholesterol levels for the two isoforms. Moreover, APOE4 also shows the highest receptor binding affinity among these three APOE variants [27].

Recently, the focus of APOE functions has transferred from amyloid plaques to tau pathology. Importantly, the APOE receptors (LDLR, LRP1, HSPG, TREM2) are also reported to be functional in tau spreading, indicating a close relationship between APOE and tau pathology. Shi et al. [28] first reported that tau P301S mice with human APOE4 knock-in (KI) show a significantly more severe neurodegenerative phenotype than that with APOE3/2 KI. Conversely, APOE knockout suppresses the progression [28]. Neuronal APOE expression, though minor (∼15% of APOE expression in the brain), is toxic to neuron. Xu et al. [29] found that without stresses, neuronal APOE expression is strongly inhibited by intron-3 retention, however, stressors such as kainic acid injection dramatically induce APOE mRNA maturation as well as protein expression. The expression of APOE, especially APOE4, increases neuronal toxicity by promoting tau phosphorylation and amyloid-β production, and depletion of neuronal APOE ameliorates tau pathology in tau P301S mouse model. Similar to APOE functions in neurons, microglial APOE is also reported to promote tau-mediated neurodegeneration. Microglia with the APOE4/4 genotype can also generate more lipid droplets, thereby inducing microglial dysfunction and tau phosphorylation in neurons [30]. Decreasing microglial APOE expression by overexpressing LDLR (low-density lipoprotein receptor) in microglia reduces tau propagation by inactivating microglia and reducing reactive astrocytes. APOE particles generated from microglia are generally larger and show neurotoxic compared to those from astrocytes [31]. It is worth noting that specifically removing astrocytic APOE4 markedly mitigates tau spreading as well as tau-induced neurodegeneration, indicating that astrocytic APOE is involved in tau-induced synaptic loss [32]. Rare APOE variants further underscore its tau-centric role: PSEN1 mutation carrier with the APOE3 R136S variant exhibits abundant Aβ plaques but strikingly attenuated tau propagation [2]. This important case suggests the essential role of APOE in tau propagation in AD. The anti-tau mechanisms of this mutation have been unraveled by different groups. APOE3 R136S shows the weakest receptor binding affinity comparing to APOE2/3/4, whereas APOE4 shows the highest interaction [27,33]. APOE3 R136S barely interacts with tau receptors, thereby disinhibiting tau uptake and degradation by microglia [33]. Similarly, it is also shown that ApoE3 R136S strongly binds tau and decreases its cellular uptake, abrogating tau pathology propagation in AD brains [34]. It is also reported that APOE3 R136S significantly mitigates tau spreading by inhibiting the microglial cGAS-STING-IFN pathway, promoting tau degradation [35]. Even APOE4 R136S mutation protects human neurons from APOE4-induced tau uptake and accumulation via HSPG (Heparan sulfate proteoglycan) [36].

### 4.2 TREM2

Triggering Receptor Expressed on Myeloid Cells 2 (TREM2) is a 230-aa glycoprotein exclusively expressed in microglia in the human brain. In 2013, TREM2 R47H variant was first identified as an LOAD (late-onset AD) genetic risk factor with the odd ratio of 4.5 by two different groups [37]. Subsequent studies identified ∼2–4-fold AD risk elevations for other TREM2 mutations (e.g., R62H, D87N) [37]. Most of these mutant TREM2s, R47H included, partially lost their ligand-binding affinity to different extents [38]. As a membrane receptor, TREM2 is reported to bind to various ligands including HDL, LDL, APOE and Aβ to activate intracellular signaling through its adaptor DNAX-activation protein 12 (DAP12/TYROBP) [39]. The important function of TREM2 is revealed by single-cell RNA sequencing that TREM2 is essential for microglial transition to disease-associated microglia (DAM)—a phagocytic state enriched near Aβ plaques in AD brains [19]. DAM are also featured by increased phagocytosis and close localization near the plaques. In addition, the major functions of TREM2 in microglia are phagocytosis of Aβ, microglial survival, microgliosis, migration and chemotaxis in microglia, which are found by different studies [38]. Intriguingly, TREM2 is also required for APOE expression in turn, thereby TREM2 and APOE together facilitating microglia activation in neurodegenerative disease [40]. Some studies also link TREM2 to inflammation, with contradictory conclusions. The anti-inflammation functions of TREM2 are observed in various mouse models. TREM2 also inhibits the transcription of TNFα, IL1β and NOS2. However, several studies also found the pro-inflammatory effect of TREM2, reflecting context-dependent duality [38]. After defining TREM2 mutation as a strong AD genetic risk factor in 2013, a large proportion of TREM2 studies try to figure out its relationship with Aβ pathology. However, inconsistent results are observed.

Only a few studies focus on TREM2 functions in tau pathology. AD patients with TREM2 R47H mutation show higher levels of both total tau and phospho-tau in CSF [41]. However, clinical evidence shows that tau spreading is positively correlated to TREM2 mRNA level or soluble TREM2 (sTREM2) in CSF. Both protective and exacerbating functions of TREM2 in tau pathology were reported by different studies. In a hTau mouse model, Bemiller et al. [42] found that TREM2 deletion accelerates tau propagation, with the reduction of microgliosis. TREM2 could also be further cleaved by α-secretase to release sTREM2. Microglia-derived sTREM2 ameliorates tau phosphorylation in neurons after up-taking by transgelin-2 [43]. Nevertheless, another study surprisingly found that in tau P301S mouse brain, TREM2 deficiency does not promote, but inhibits neurodegeneration and microgliosis. No significant difference in phosphorylated tau spreading was observed [44]. Furthermore, Sayed et al. [46] reported that TREM2 knockout alleviates tau-mediated microglial activation and brain atrophy, however, TREM2 haploinsufficiency promotes the expression of proinflammatory markers and accelerates brain atrophy at the late stage [45]. Moreover, TREM2 R47H tau P301S mouse shows reduced microglia-mediated neurodegeneration. Activation of TREM2 by an activating antibody promotes microgliosis and tau spreading [47]. Therefore, due to inconsistent results observed, the effect of TREM2 on tau pathology may be dose- and stage-dependent [48]. At early stages, TREM2 or sTREM2 may have beneficial functions. However, at late stages, the expression of TREM2 may greatly facilitate tau pathology in AD. More in-depth studies are certainly required to iron out the complicated relationship between TREM2 and tau pathology.

### 4.3 Tau kinases

Phosphorylation is the most well-known and important post-translational modification of tau, occurs at over 80 sites, critically regulating its aggregation propensity and microtubule dissociation [1]. GSK3, CDK5, PKA and casein kinase 1 are the major tau kinases, and PP2A is well-studied as a crucial tau phosphatase [49]. Tau phosphorylation also promotes its proteolytic cleavage by caspases, producing tau fragments with increased propensity of aggregation [50]. Cytokines such as IL-1β and IL-6 promote tau phosphorylation via p38/MAPK or GSK3β. Glycogen synthase kinase-3 (GSK-3) is considered as the major tau kinase, ubiquitously expressed for cell metabolism and signaling. Unusual activation of GSK-3 is associated with AD, and upregulation of GSK-3 expression is found in postmortem human AD brains. In the tau transgenic mouse model, kinase inhibitor treatments block tau phosphorylation and thus reduce tau aggregates. However, inhibition of tau phosphorylation also leads to severe tau abnormalities, indicating the important physiological role of tau phosphorylation [50]. Several GSK3 inhibitors have entered clinical trials to treat AD or other rare tauopathies. Lithium, a non-specific GSK3 inhibitor, though showed insignificant effects in some trials, it reduces tau phosphorylation and enhances memory performance in patients with mild cognitive disorders in a small trial [50]. Another GSK3 inhibitor, Tideglusib (NP-12), also entered clinical trials.

### 4.4 Tau receptors

The process of extracellular tau uptake by neurons or glial cells is essential for tau toxicity and propagation. The major receptor for tau is Low-density lipoprotein receptor-related protein 1 (LRP1) [51]. LRP1 is a large membrane protein (600 kDa), which belongs to the low-density lipoprotein receptor (LDLR) family. Depletion of LRP1 reduces tau uptake in IPSC-derived neurons, and LRP1 downregulation inhibits tau spreading in mouse models. Heparan sulfate proteoglycans (HSPGs) are also important tau receptors for tau aggregates binding and uptake. Moreover, there are also other tau receptors identified. Tau may enter cells via muscarinic receptors (M1 and M3) or direct endocytosis as well [1]. Additionally, Aβ-induced tau uptake also depends on its physical interaction with fibroblast growth factor receptor 3 (FGFR3) [52]. Therefore, these tau receptors are essential for tau spreading and are possible future therapeutic targets developed to treat tau pathology.

### 4.5 E3 ubiquitin ligases

Tau is predominantly degraded via the ubiquitin-proteasome system (UPS) rather than autophagy, with E3 ubiquitin ligases playing a central role in tagging misfolded tau with polyubiquitin chains for proteasomal clearance. There are tremendous identified ubiquitylation sites of tau according to different studies and databases. These ubiquitylation sites includes K254, K259, K267, K274, K281, K290, K294, K298, K311, K317, K321, K331, K340, K353, K370, K491, K571, K598, K607, K608, K660, K670, K686.

The most well-known E3 ligase for tau protein is CHIP (carboxyl terminus of the Hsc70-interacting protein). Tau is recognized and ubiquitylated by CHIP in a tau phosphorylation-dependent manner [53]. The overexpression of CHIP could rescue phosphorylated tau-induced cell death. The following studies unravel that CHIP binds to tau at three primary binding sites within the repeat domains, with the 308–315 domain as the major binding site. Treadmill exercise could improve cognitive ability in APP/PS1 transgenic mice by promoting E3 ubiquitin ligase (UCHL-1 and CHIP) to remove Aβ and phosphorylated tau [54]. TRIAD3A also interacts with and ubiquitylates tau [55]. Overexpression of TRIAD3A in the tau mouse model notably reduces tau phosphorylation and aggregation. Disease-associated mutation of TRIAD3A (R694C) increases gliosis and tau aggregation in vivo. MARCH7 (Axotrophin) was another identified tau-binding E3 ligase [56]. MARCH7 Ring-variant domain mono-ubiquitinates tau protein at multiple sites including the microtubule-binding domain, which diminishes its microtubule-binding. Interestingly, MDM2, the most famous E3 ligase for tumor suppressor P53, is also reported to bind to the microtubule-binding domain of Tau [57]. Therefore, the MDM2-dependent P53 ubiquitination is inhibited by tau, however, whether MDM2 also promotes the ubiquitylation of tau still requires further studies. Given the importance of E3 ligase and ubiquitylation on tau pathology, RING-based degraders are also developed to selectively ubiquitylate and degrade misfolded tau proteins [58]. These approaches could prevent or reverse tau pathology both in vitro and in vivo, providing therapeutic potential for the many disorders driven by intracellular protein aggregation.

### 4.6 AEP

**Fig. 2:**
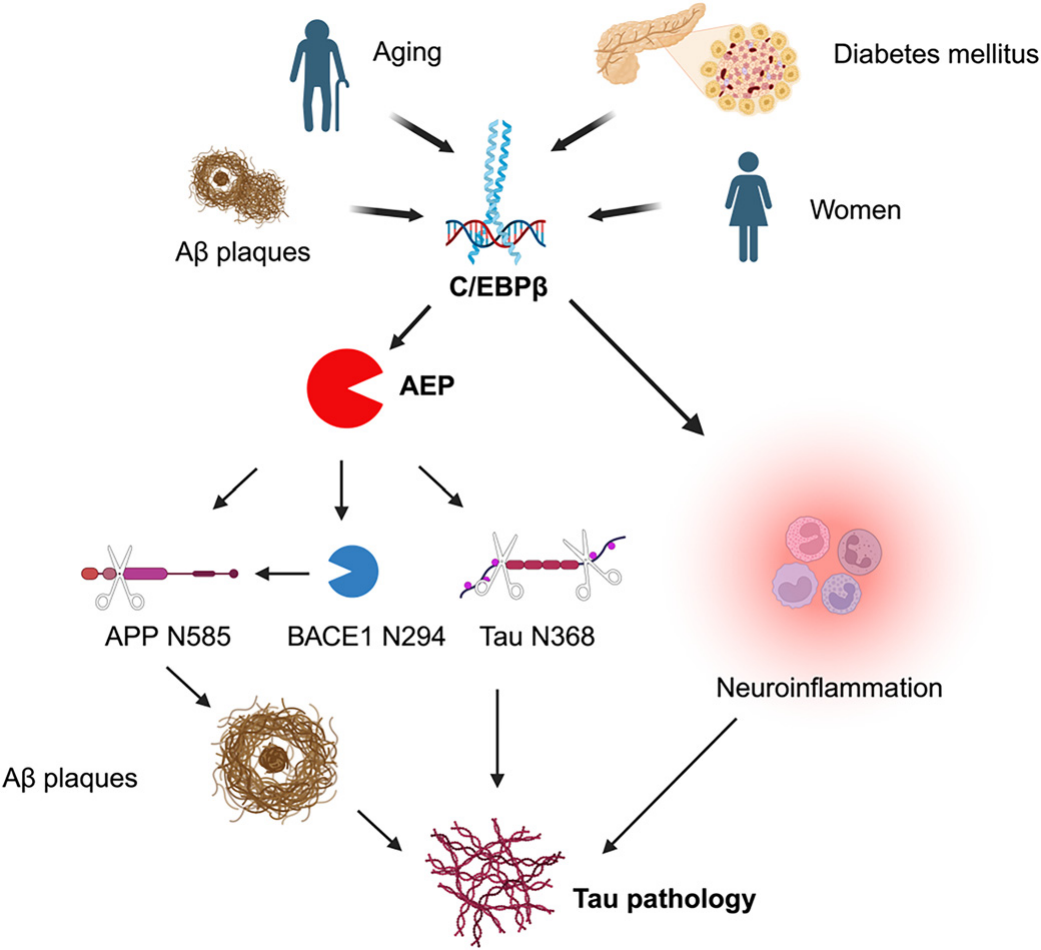
**C/EBPβ/AEP signaling in tau pathology in AD.** C/EBPβ can be activated by various driving forces for tau pathology in AD, including but not limited to aging, Aβ plaques, diabetes mellitus and gender (women). Consequently, AEP expression is transcriptionally upregulated by C/EBPβ to produce truncated tau fragments with higher aggregability. Moreover, AEP cuts APP and BACE1 to accelerate Aβ production, thereby facilitating tau spreading in AD. Additionally, active C/EBPβ also elevates neuroinflammation to promote tau pathology. This schematic was created using Biorender (http://biorender.com).

Proteolytic processing plays a critical role in driving tau aggregation and propagation [59]. Asparaginyl endopeptidase (AEP, also known as legumain) is the major cysteine protease in the lysosome by cleaving the substrates at the C-terminus of asparagine residues. AEP expression increases with age or Aβ pathology in both human and mouse brains [6]. A landmark study in 2014 by Zhang et al. [59] reported that AEP cuts tau at both N255 and N368 residues that flank the repeat domains of tau in human AD brain in an age-dependent manner, processing tau into a repeat domain-enriched, more aggregable fragment. Notably, AEP cleavage stimulates tau hyperphosphorylation, whereas tau phosphorylation does not affect AEP fragmentation of tau. Interestingly, an additional tau cutting site by AEP is identified at N167 in microglia, increasing its property of aggregation as well [60]. These findings position AEP as a dual-compartment regulator of tau proteostasis, driving both neuronal and microglial tau fragmentation to exacerbate propagation and toxicity **(**Fig. 2**)**. Notably, Genetic AEP ablation in tau P301S mice markedly reduces tau burden and rescues cognitive deficits [59]. Moreover, AEP also processes amyloid precursor protein (APP) at N373/N585, priming it for β-secretase (BACE1)-mediated cleavage and Aβ generation [61]. Interestingly, AEP also enhances the enzymatic activity of BACE1 by cutting BACE1 at N294 [62]. These dual roles in tau and Aβ pathways underscore AEP’s centrality in AD pathogenesis. Therefore, an orally active and brain-penetrant AEP inhibitor Compound #11A was developed to specifically inhibit the activity of AEP, and it currently undergoes pre-clinical studies [63]. Inhibition of AEP by either genetic knockout or inhibitor in AD mouse models decreases the tau/Aβ pathology and neurodegeneration [64,65].

### 4.7 C/EBPβ

CCAAT/enhancer binding protein-β (C/EBPβ), a ubiquitously expressed transcription factor of the basic-leucine zipper family, regulates genes involved in inflammation, differentiation, metabolism, and immune responses [6]. C/EBPβ was originally identified as IL6DBP/NFIL6 for its role in interleukin-6 signaling. In mammals, C/EBPβ is a unique gene with no intron, and C/EBPβ mRNA is the first identified mRNA to translate three different proteins: LAP1, LAP2 and LIP, respectively. LAPs are generally considered as transcriptional activators. In contrast, LIP, lacking the transcription activating domain, is believed to act as a transcriptional repressor by competitive inhibition. C/EBPβ expression increases with age in the human brain but is suppressed in long-lived individuals (> 90 years) [66]. It ranks among the top age-dependent microglial (ADEM) transcription factors [67]. Remarkably, C/EBPβ is dramatically elevated in the human AD brains compared to the healthy brains. Inflammation and oxidative stress are also well-known triggers for the activation of C/EBPβ [68]. Since C/EBPβ can be activated by various driving forces of tau pathology in AD, accordingly, numerous studies report that C/EBPβ drives AD progression including tau pathology by different mechanisms **(**Fig. 2**)** [6]. C/EBPβ is a well-established transcription factor for AEP, cleaving various key pathological proteins such as Tau and APP to accelerate neurodegeneration [59,61]. C/EBPβ also dictates MAPT mRNA expression and plays a critical role in tau accumulation and neurodegeneration [69]. In microglia, C/EBPβ also regulates the pro-inflammatory gene expression. As a matter of fact, microglial-specific depletion of COP1, a glucose-sensing E3 ligase for C/EBPβ, leads to C/EBPβ accumulation in microglia, promoting tau pathology and neurodegeneration [[70], [71], [72]]. Specifically, C/EBPβ activates microglial complement pathway to induce neuronal toxicity. Furthermore, C/EBPβ is the crucial transcription factor for APOE, favorably promoting the expression of APOE4 variant, which is the most well-known genetic risk factor for AD [73]. APP fragment by AEP cleavage and APOE4 also strongly activate C/EBPβ expression in return, creating a self-reinforcing loop that accelerates neurodegeneration[69]. Gut dysbiosis-derived metabolites (e.g., 12-HHTrE, PGE2) activate the C/EBPβ/AEP axis in the brain, accelerating Aβ/tau pathology. Antibiotic treatment attenuates this signaling, rescuing cognition in AD models [74]. Antibiotic treatment diminishes this signaling and improves cognitive functions in 5xFAD. Women’s elevated AD risk (4-fold higher incidence) is partly mediated by follicle-stimulating hormone (FSH), which activates C/EBPβ/AEP to drive tau/Aβ accumulation [12,13]. Therefore, these findings strongly support that C/EBPβ/AEP signaling drives AD pathogenesis. According to this theory, a neuronal specific Thy1-ApoE4/C/EBPβ double transgenic mouse model without any rare human mutations was developed as AD sporadic model that develop both Aβ and tau pathology with mouse endogenous machinery, providing a huge potential for the sporadic AD studies [48]. ST101, a 38 amino acid synthetic peptide of d-amino acids, is a potent antagonist of C/EBPβ [75]. ST101 is currently being evaluated in patients with refractory solid tumors (including glioblastoma) in the Phase 2 clinical trial. Therefore, ST101 has huge potential to treat AD with modifications. These observations support that C/EBPβ/AEP pathway may also drive aging, the major risk factor for AD. Consequently, C/EBPβ/AEP pathway is also age-dependently activated to promote different age-dependent diseases’ progression including AD, PD, Cancer, Arteriosclerosis and Diabetes.

In conclusion, diverse proteins steer tau pathology in AD. Briefly, APOE, especially the APOE4 variant, impairs the functions of neurons and microglia to drive tau pathology. TREM2 overactivates microglia to facilitate tau spreading and neuroinflammation. Tau kinases (e.g., GSK3β, CDK5) and tau receptors (e.g., LRP1, HSPGs) drive phosphorylation and trans-synaptic spread of pathological tau, respectively. Phosphorylated tau could also be ubiquitylated by E3 ligases such as CHIP for proteasomal degradation. Moreover, the C/EBPβ/AEP pathway could be activated by diverse major tau drivers to enhance tau truncation, aggregation and spreading. Therefore, targeting these key proteins is essential for the treatment to reverse the cognitive decline in AD patients.

## 5 Conclusion and perspectives

For decades, Aβ plaques were considered the primary driver of neurodegeneration in AD. However, emerging clinical evidence point out that tau pathology, but not Aβ plaques, exhibits a stronger correlation with cognitive dysfunction and brain atrophy. Therefore, tau pathology draws increasing attention in the field of AD. In this review, we summarize the major identified factors driving tau pathology in AD progression. The major physiological driving force is aging. Besides, gender (woman) also alters the progression. Aβ, diabetes are key pathological risk factors for tau pathology in AD. Different cell types in the brain are involved in the prion-like pathogenic tau spreading. Pathological tau fibrils propagate between neurons and the toxicity elicits neuronal dysfunction by different mechanisms. In addition, microglia and microglia-dependent cytotoxic T cell infiltration enhance tau spreading, whereas the function of astrocytes in tau pathology is less studied and remains controversial. Multiple central molecular regulators are implicated in tau pathology in AD. The most well-known AD risk protein, APOE, especially APOE4 variant, accelerates tau pathology, irrespective of its derivation from neurons, microglia or astrocytes. The other important AD risk protein, TREM2, amplifies tau propagation via microglial activation. Notably, Tau kinases (e.g., GSK3β) enhance phosphorylation and aggregation, while tau receptors (e.g., LRP1) mediate extracellular tau uptake. However, phosphorylated tau could be recognized by E3 ligase CHIP for proteasomal degradation, thereby preventing tau spreading. Furthermore, C/EBPβ/AEP pathway, activated by various stimulatory factors, drives tau truncation and Aβ production, forming a self-reinforcing loop. Although these factors are discussed separately, they may intimately function as a network to jointly promote the progression of tau pathology. Some of the factors somehow seem irrelevant at present, but they are interweavingly correlated with each other, although the mechanisms require future investigation.

Several therapeutic strategies targeting tau pathology in AD have been developed (Table 2). One direct strategy is targeting tau aggregation by immunotherapy. TRx-237 was the only anti-tau drug in the phase III clinical trial in 2022 but failed. No significant cognitive restoration was observed in the trial, although it showed effective for NFTs clearance. More precise patient stage division and multi-drug combined therapy may be helpful to improve its performance. Up to now, direct anti-tau drug failures stack up and none of them are approved by FDA to treat AD. Based on the above discoveries, there are also other potential therapeutic strategies. Lecanemab and Donanemab are FDA-approved drugs to treat AD by effectively targeting Aβ plaques. They could theoretically inhibit Aβ-induced tau pathology. However, their effects on tau pathology have not been evaluated yet and severe side effects are reported in the treated patients. Dulaglutide is a GLP1R agonist, which is originally developed to treat T2DM. It also shows a protective effect on reducing cognitive dysfunctions in the clinical study [11]. Besides, Lithium, Tau kinase GSK3 inhibitor reduces tau phosphorylation and improves memory performance in a small trial [50]. MAPT Rx, a Tau-specific antisense oligonucleotide (ASO) reduces tau in the brain and cerebrospinal fluid in tau transgenic mice, reversing the preexisting tau pathology and tau seeding activity [76].

**Table 2:** **Overview of different potential drugs for tau pathology in AD**.

Drug name(s)	Drug target(s)	Key mechanisms	Status	Reference(s)
TRx-237	Tau protein	Directly target tau protein as antibody	Failed in phase III clinical trial	[79]
Lecanemab and Donanemab	Aβ plaques	Inhibit Aβ-induced neuronal death and tau pathology	FDA-approved; Delay congnitive decline	[80]
Dulaglutide	GLP1R agonist	Elusive	Reduce cognitive dysfunctions in a large trial	[11]
Lithium	Tau kinase GSK3	Inhibit Tau phosphorylation	Improve memory performance in a small trial	[50]
MAPT Rx	Tau	Knocking down tau expression	Pass phase I clinical trial	[76]
FSH-blocking antibody	FSH	Inactivate C/EBPβ/AEP pathway	Undergo Pre-clinical studies	[12,13]
#11a	AEP	Inhibit AEP-depedent tau cleavage and aggregation	Undergo Pre-clinical studies	[63]

There are also other potential therapeutic targets, albeit awaiting clinical trials for validation. For instance, FSH-blocking antibody reverses tau/Aβ neuropathology and cognitive decline in AD mouse model, suggesting a potential AD therapy, especially for aged women with high FSH levels [13]. TREM2 activating antibodies are also developed to treat AD, however, contradictory results were observed due to the complex functions of TREM2 and microglia in AD [47]. Noticeably, AEP specific inhibitor, #11a, blocks tau/Aβ pathology in AD mouse models by inhibiting both tau aggregation and Aβ production [64,65]. Moreover, based on these findings, A CRISPR-based gene editing therapy could be developed to precisely target and modify these driving factors to halt tau pathology. While progress has been made, continued research is imperative to unravel the complexity of tau pathology. Combination therapies targeting multiple pathways or leveraging precision medicine (e.g., patient stratification by disease stage) may hold transformative potential. By integrating mechanistic insights with innovative therapeutic design, the field moves closer to halting tau-driven neurodegeneration in AD.

## CRediT authorship contribution statement

**Yifan Luo:** Writing – review & editing. **Honglu Yu:** Writing – review & editing. **Keqiang Ye:** Writing – review & editing.

## Declaration of competing interest

The authors declare that they have no conflicts of interest in this work.
